# Role of Carbonyl Modifications on Aging-Associated Protein Aggregation

**DOI:** 10.1038/srep19311

**Published:** 2016-01-18

**Authors:** Maya Tanase, Aleksandra M. Urbanska, Valerio Zolla, Cristina C. Clement, Liling Huang, Kateryna Morozova, Carlo Follo, Michael Goldberg, Barbara Roda, Pierluigi Reschiglian, Laura Santambrogio

**Affiliations:** 1 Department of Pathology, Albert Einstein College of Medicine, 1300 Morris Park Avenue, New York, 10461, USA; 2 Department of Chemistry, University of Bologna, Via Selmi 2, 40126, Bologna, Italy; 3 by Flow srl, Via Fani 11/b, 40127 Bologna, Italy; 4 Department of Microbiology and Immunology, Albert Einstein College of Medicine, 1300 Morris Park Avenue, New York, 10461, USA

## Abstract

Protein aggregation is a common biological phenomenon, observed in different physiological and pathological conditions. Decreased protein solubility and a tendency to aggregate is also observed during physiological aging but the causes are currently unknown. Herein we performed a biophysical separation of aging-related high molecular weight aggregates, isolated from the bone marrow and splenic cells of aging mice and followed by biochemical and mass spectrometric analysis. The analysis indicated that compared to younger mice an increase in protein post-translational carbonylation was observed. The causative role of these modifications in inducing protein misfolding and aggregation was determined by inducing carbonyl stress in young mice, which recapitulated the increased protein aggregation observed in old mice. Altogether our analysis indicates that oxidative stress-related post-translational modifications accumulate in the aging proteome and are responsible for increased protein aggregation and altered cell proteostasis.

Protein aggregation is a general terminology used to describe the association of proteins into larger assemblies following loss of secondary, tertiary or quaternary structure and often loss of biological activity^1^. Protein aggregation is a common biological phenomenon associated with the inability of the cell to maintain homeostasis of the proteome (proteostasis)^1^. Under physiological conditions, the tendency of *de novo* synthesized unfolded proteins to aggregate is regulated by several chaperones that aid in their folding^2^,^3^. Soluble aggregation is also commonly observed in ubiquitinated unfolded proteins before proteasome degradation or in damaged proteins before translocation into lysosomes by chaperone-mediated autophagy^4^,^5^,^6^. Additionally, temporal changes to cellular homeostasis (temperature, pH, water content and salt/ions concentration) can induce transitory protein unfolding and soluble aggregation^1^.

During pathological conditions, protein aggregation is a common occurrence giving rise to the group of diseases collectively known as protein conformational diseases. In many degenerative diseases of the CNS, such as Alzheimer’s, Parkinson’s and Huntington’s disease protein aggregation is a common pathological hallmark due to amino acid mutation and changes in the primary structure of the proteins^7^,^8^,^9^,^10^,^11^.

Size-wise, aggregates can range considerably, from protein oligomers up to visible cytosolic inclusions, known as the aggresome^12^. The sub cellular location of these aggregates can also vary, from perinuclear to peri-endolasmic reticulum (ER) or intra-endosomal. Perinuclear aggregates (aggresomes) co-localize with the microtubule organizing center and mostly contain terminally aggregated proteins^12^, whereas ER-associated aggregates are mostly formed by soluble aggregates of ubiquitinated misfolded proteins^3^ and endosomal aggregates are inclusions that co-localize with autophagic markers^13^,^14^,^15^. The functional consequences of aggregation are also variable, from up-regulation of autophagy^16^, to cellular apoptosis due to aggregate-related cytotoxicity.

Recently, it has been reported that during physiological aging proteostasis gradually becomes compromised and several hundred proteins tend to become more insoluble and aggregate^17^,^18^. A few of these proteins have been shown to have common biochemical and biological properties, such as a primary structure with amino acids stretches often found in proteins associated with neurodegenerative diseases and a secondary structure with increased beta-sheets^17^. However, for the majority of other proteins whether there is a tendency to aggregate in aging cells is still unclear.

The aging process is known to be associated with increased oxidative stress, which induces post-translational modifications of proteins including glycation, glycoxidation, lipoxidation and carbonylation. We previously mapped several of these modifications in aging bone marrow and splenic immune cells^16^. These modifications are also often observed in diseases such as diabetes, metabolic syndrome, chronic inflammation and degenerative conditions. The aim of this study was to determine whether post-translational modifications associated with aging-related oxidative stress, could be responsible for increased protein aggregation. To this goal, we separated protein aggregates from bone marrow and spleen of 3, 12 and 22 month old mice using hollow fiber field-flow fractionation (HF5) coupled with UV and multi-angle light scattering (MALS) detection^19^. Subsequent to their separation according to their hydrodynamic size (and molecular weight), we analyzed their biochemical composition by mass spectrometry. Data indicate a clear association between carbonylation of the cellular proteome and age-associated increased aggregation. This finding was further confirmed by the increased aggregation of the cellular proteome of young mice following *in vivo* induction of protein carbonylation.

## Results

### *In Vivo* analysis of oxidative stress

**Figure 1:**
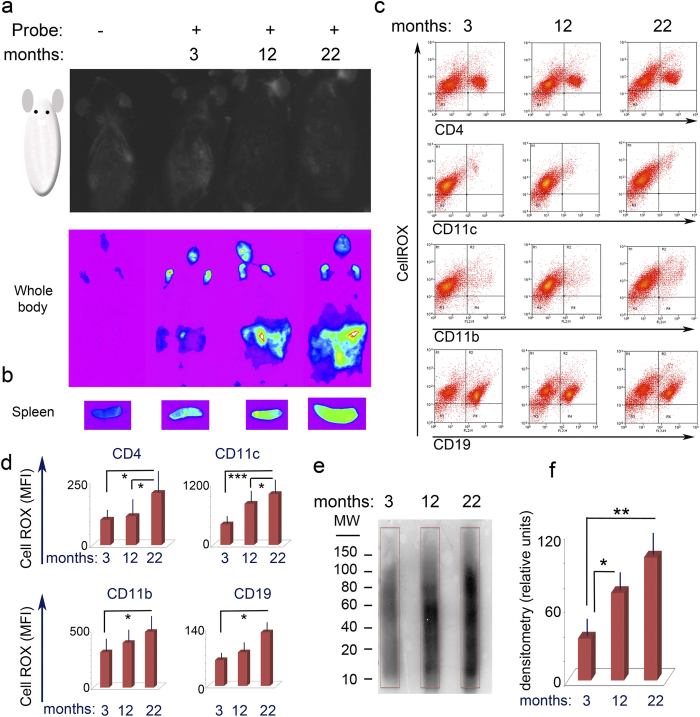
*In vivo* detection of oxidative stress in aging mice. **(a)** *In vivo* Detection of Oxidative Stress in 3, 12 and 22 months old mice. Mice were injected i.v with 2.5 mM of the CellROX probe which fluoresces upon binding to reactive oxygen species. Fluorescence was imaged using the *In-Vivo* Imaging System FX PRO (Carestream). One out of three experiments is shown. Each experiment included two independent mice for each age group, is shown. (**b)** *Ex vivo* detection of oxidative stress in spleens harvested from the same mice imaged in (**a**). (**c)** FACS analysis of splenic cell subpopulations isolated *ex vivo*, following CellROX injection in the same mice imaged in (**a**). **(d)** Bar graphs representing CellROX mean fluorescence index of splenic cell subpopulations in 3, 12 and 22 months old mice. Average and standard deviation calculated from three separate experiments, each experiment included two independent mice for each age group. *p < 0.05 **p < 0.01 (**e)** Western blot analysis of total protein carbonylation detected in splenic cell lysates harvested from the 3, 12 and 22 months old mice described in (**d**). One out of three blots is shown. Quadrangles indicate area scanned for densitometric analysis. (**f)** Densitometric analysis of total protein carbonylation as detected by western blot analysis. Average and standard deviation calculated from three separate western blots.

Aging-related oxidative stress could be a potential source of an increase of carbonyl-type post-translational protein modifications. Thus, as a first step we determined whether increased oxidative stress could be observed *in vivo* in aging mice. To detect *in vivo* production of reactive oxygen species (ROS), we optimized a method commonly used *in vitro* to measure cellular ROS. The CellRox probe, which fluoresces upon binding to reactive oxygen species, was injected i.v. into 3, 12 and 22 month old mice and total body fluorescence was analyzed after 15 minutes by *in vivo* fluorescence imaging. An age-dependent increase in fluorescence was observed among the three age groups (Fig. 1a). Fluorescence was more readily visualized in parenchymal organs located in the abdominal cavity due to easier detection of the fluorescence signal, as compared to organs protected by bone structures (Fig. 1a). The results indicate an increase of “*in vivo*” oxidative stress in aging mice. To confirm further the *in vivo* fluorescence imaging data, spleen and bone marrow were harvested *ex vivo* from the CellRox injected mice and imaged. As expected, an age-dependent increase in fluorescence was also detected in the isolated organs (Fig. 1b). To quantify at the cellular level the amount of oxidative stress, single cell suspensions of *ex vivo* harvested splenic cells were stained with antibodies specific for CD4^+^ T cells, B cells, macrophages and dendritic cells (DC) (Fig. 1c and Supplement Fig. 1). Increased CellRox fluorescence could be detected in all cellular sub-populations purified from the aging mice when compared to 3 and 12 month old animals (Fig. 1c,d). As expected, macrophages and DC presented with the highest amount of oxidative stress due to the higher levels of ROS and reactive nitrogen species (RNS) normally produced by these cells (Fig. 1c,d). Similarly, age-dependent increases in fluorescence were also observed in bone marrow cell subpopulations (Supplement Fig. 1). To determine whether age-related increases in oxidative stress correlated with increased protein carbonylation, spleen lysates were run on an SDS-PAGE and the membrane probed with an antibody, which detects dinitrophenylhydrazine (DNPH)-derivatized carbonyl groups^16^. An age-dependent increase in protein carbonylation was determined by western blot analysis (Fig. 1e,f). Taken together the data indicate enhanced free radical production and protein carbonylation in 22-month-old mice as compared to 3 and 12-month-old mice.

### Biophysical analysis of Aging-associated Protein Aggregates

**Figure 2:**
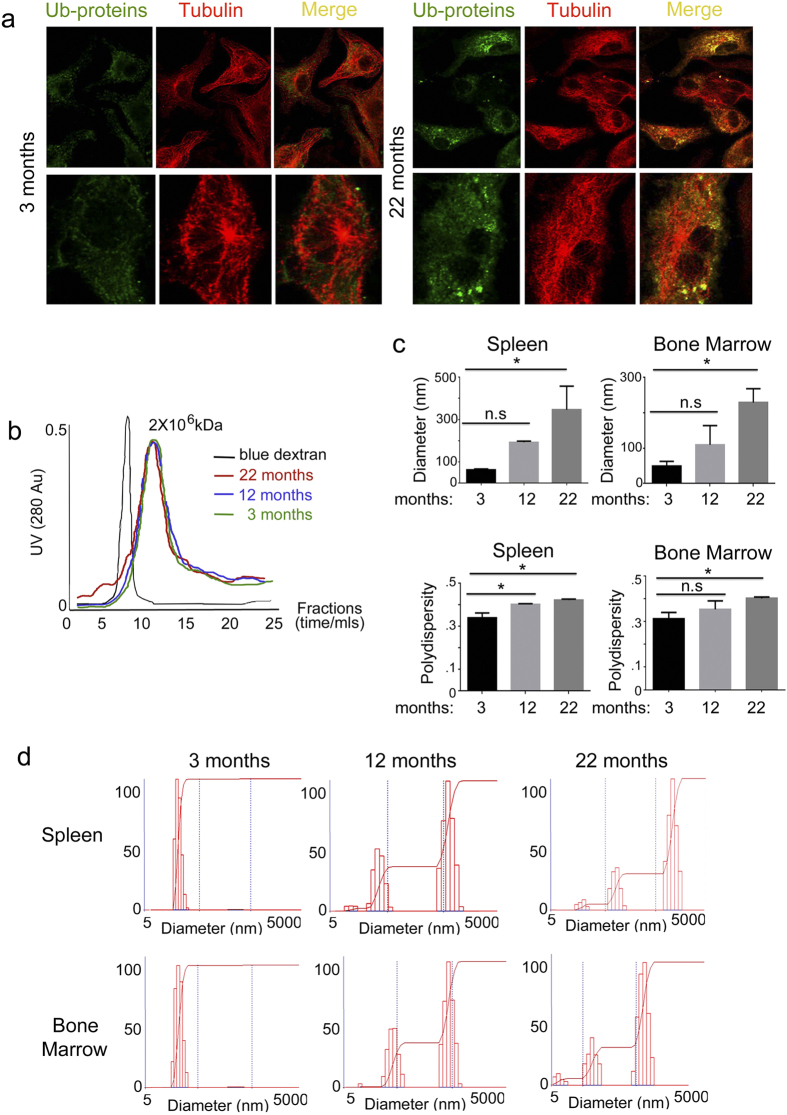
Characterization of protein aggregates in bone marrow and spleen from 3 12 and 22 months old mice. **(a)** Immunofluorescence analysis of ubiquitinated-protein aggregates (Ub-proteins), associated with tubulin filaments in CD34^+^ bone marrow cells collected from 3, 12 and 22 months old mice. (**b)** Size exclusion chromatography of CD34^+^ bone marrow total protein lysates collected from 3, 12 and 22 months old mice. One out of three runs is shown; each run was performed using lysates from three independent mice from each age group. (**c)** Mean diameter and polydispersity of protein aggregates present in the bone marrow and spleen total cell lysates collected from 3, 12 and 22 months old mice. Average and standard deviation calculated from three separate experiments, each experiment included independent mice for each age group. (**d)** Dynamic light scattering measurement of protein aggregates present in the bone marrow and spleen of total cell lysates collected from 3, 12 and 22 months old mice. One out of three experiments is shown. Each experiment included independent mice for each age group.

As a next step we set to investigate whether aging-related oxidative stress and associated protein modifications could induce increased aggregation of the aging proteome. Unfolded proteins often assemble in larger aggregates, that can be visualized as ubiquitinated cytosolic inclusions, known as the aggresome, associated with the cellular cytoskeleton^12^. To detect these aggregates CD34^+^ bone marrow cells, purified from 3, and 22-month-old mice, were stained for dynamin and ubiquitin. Large ubiquitin positive and tubulin positive aggregates could be visualized in aging bone marrow CD34^+^ cells but not in the same cells isolated from young mice (Fig. 2a).

Fast protein liquid chromatography is also often used to separate very large protein aggregates, in the million-kDa range, that can easily be distinguished from the cellular un-aggregated proteome. Thus, to further analyze the aggregated proteome, cell lysates, collected from 3, 12 and 22 months old bone marrow CD34^+^ cells were FPLC fractionated. Large aggregates, which eluted after the void volume and above the blue dextran marker (2 × 10^6^ kDa), could be separated in the lysate from 22 months old CD34^+^ bone marrow cells but not in the 12 months and 3 months old cell lysate (Fig. 2b). However one of the constrains of using immunofluorescence as well as FPLC fractionation is that both techniques can only visualize very large aggregates and cannot resolve a large dynamic of molecular weights. Thus, total cell lysates from spleen and bone marrow collected from 3, 12 and 22 months aged mice, were analyzed by dynamic light scattering which can resolve protein aggregates ranging from nanometers (monomer and low-order oligomer) to micrometers (high-order aggregate and aggregate particles) in diameter. A wider range of protein aggregates could be observed in 12 and 22 months old bone marrow and spleen cells lysates as compared to 3 months old (Fig. 2c–e); whereas in the 3 months old lysates the average diameter of the cellular aggregates was around 50–60 nanometer in the 12 and 22 month old lysates the diameter increased to 109–193 and 229–345 respectively (Fig. 2c–e). The aggregates degree of polydispersity also increased with age, indicating a statistically significant broader range of aggregate sizes and conformations (Fig. 2c–e).

**Figure 3:**
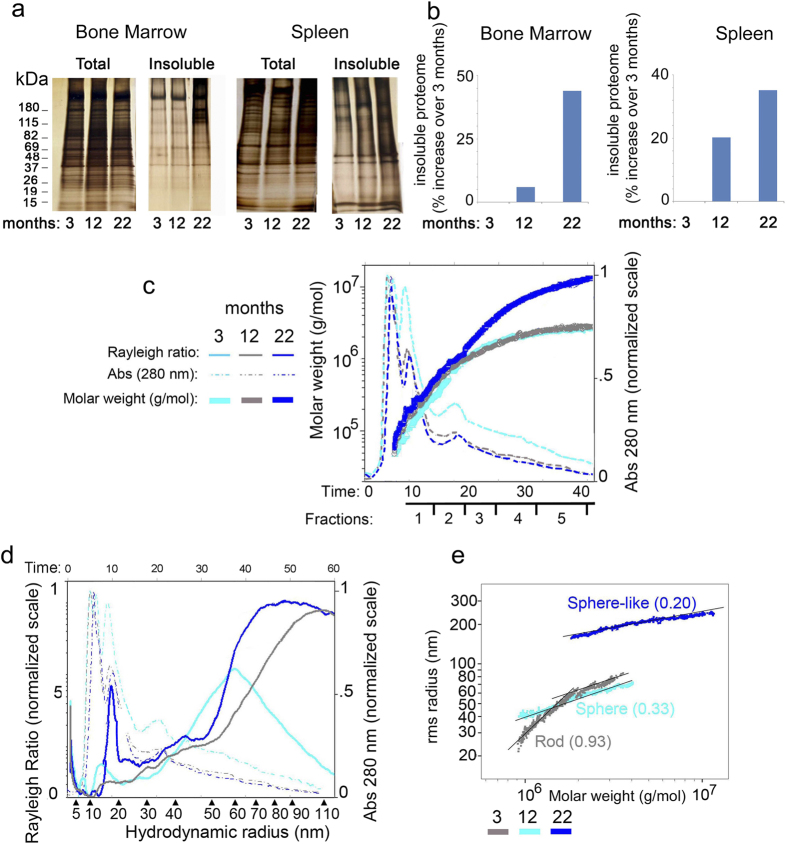
Characterization of protein aggregates in bone marrow and spleen lysates from 3 12 and 22 months old mice. **(a)** Silver staining of bone marrow and spleen, detergent soluble and insoluble total protein lysates, prepared from 3, 12 and 22 months old mice. One out of three experiments is shown; each experiment was performed using cell lysates from independent mice in each age group. (**b**) Quantification of the detergent insoluble proteome in 12 and 22 months old bone marrow and spleen lysates (calculated as percentage increase over 3 months old lysates). One out of three experiments is shown (**c)** HF5-UV-MALS of CD34^+^ bone marrow lysates collected from 3, 12 and 22 months old mice. Overlaid fractograms: dash-dotted lines report OD reading at 280 nm (right y axis); full thick lines report calculated molecular weight, displayed in logarithmic scale (left y axis); x axis reports the retention time and schematic of fraction collection. One out of four runs is shown; each run was performed using cell lysates from independent mice in each age group. **(d)** HF5-UV-MALS of CD34^+^ bone marrow lysates collected from 3, 12 and 22 months old mice. Overlaid fractograms: dash-dotted lines report OD reading at 280 nm (right y axis); thin full lines report Rayleigh ratios at 90° (left y axis); x axis reports hydrodynamic radius values. One out of four runs is shown; each run was performed using cell lysates from independent mice in each age group. (**e)** Correlation plots: rms radius values plotted against the corresponding MW values (both in logarithmic scale) of protein aggregates in bone marrow cells collected from 3, 12 and 22 months old mice. Numbers in parenthesis correspond to the slope of each plot; each value was assigned to a known conformation type.

Protein aggregates are known to remain insoluble in strong detergent buffers but can be solubilized in urea and separated by SDS-PAGE^17^. Thus, to further quantify age-related changes in protein solubility, bone marrow and spleen lysates were fractionated into detergent soluble and insoluble proteome^17^ (Fig. 3a). An increased level of insoluble proteome was observed in aging spleen and bone marrow (Fig. 3a). More specifically, an increase of almost 50% in the detergent insoluble aggregated proteome was observed in the 22 months old mice, in both spleen and bone marrow cell lysate (Fig. 3b)^19^.

To better characterize the cellular aggregates, bone marrow lysates were submitted to HF5-UV-MALS analysis, an online coupling technique employed to separate proteins according to their *diffusion coefficient* (*D*), which correlates with the protein *hydrodynamic* size, molecular weight (MW), and molecular conformation^18^,^19^. Differently from immunofluorescence and FPLC this technique allows characterizing aggregates of different molecular sizes. Moreover, in addition to dynamic light scattering the HF5-UV-MALS allows the collection of different aggregates for further analysis. HF5-UV-MALS confirmed the increased amount of high MW protein aggregates in the 22 month old bone marrow proteome as compared to the 3 or 12 month old ones (Fig. 3c, Table 1). All three bone marrow samples contained a common MW range of proteins and protein aggregates (up to 4.4 × 10^6 ^g/mol); additionally, only the 22 month old mice presented protein aggregates with MW up to 1 × 10^7 ^g/mol (Fig. 3c, Table 1).

Importantly, aggregates with the same molecular weight but collected from different ages, eluted at different retention times due to differences in their hydrodynamic size/radius (Fig. 3d,e, Table 1). These size differences between protein aggregates with the same MW value (but collected from mice of different ages: 3, 12 and 22 months) are very likely caused by changes in the aggregate molecular conformation – as previously observed for Paraquat-treated cell lysates^19^. Indeed, the conformational analysis (Fig. 3e) showed that aging-related protein aggregates in the 22 month sample only presented large *sphere-*like, highly compact conformations, whereas aggregates found in 12 months old bone marrow presented with much smaller sphere-like conformations. A more relaxed and flexible rod-like conformation was observed in 3 months old bone marrow. Altogether, our analysis confirmed the increased tendency of proteins to aggregate during physiological aging^17^,^18^ and extended our knowledge on the molecular weight, size and conformation of these aggregates.

### Biochemical Analysis of Aging-associated Protein Aggregates

**Figure 4:**
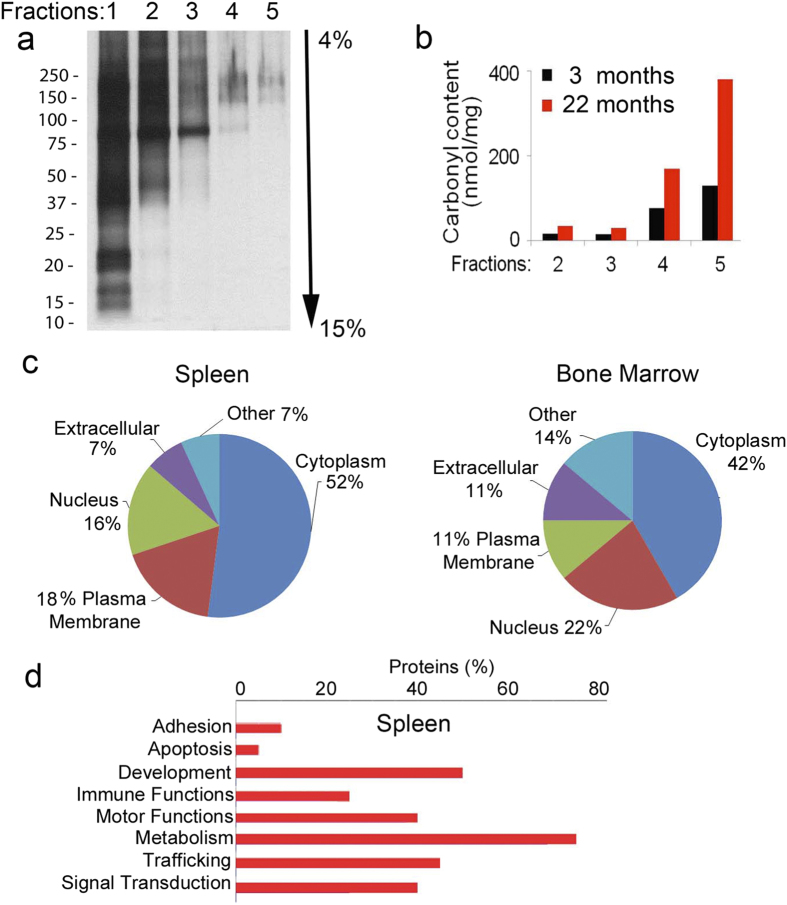
Characterization of protein aggregates in bone marrow and splenic cell lysates from 22 months old mice. (**a)** Silver staining of an SDS-PAGE of protein fractions collected from bone marrow cell lysates (22 months old mice) HF5-fractionated. One out of two gels is shown. **b)** Carbonyl content of protein fractions collected from bone marrow cells lysates HF5-fractionated as in (**a**). One out of 6 separate collections is shown. (**c)** Pie charts of the spleen and bone marrow aggregated proteomes collected from fractions 4 and 5 (as shown in (**a**). Data are compiled from two separate collections. (**d)** Histogram representation of the major functional pathways represented in the aggregated proteomes of both bone marrow and spleen and generated with the GO annotation algorithm built in Scaffold.

It has been recently reported that during physiological aging several hundred proteins tend to become more insoluble and aggregate (metastable proteins). Few of these proteins present biochemical communality known to be aggregation-prone such as poly-Q amino acid stretches or increased beta-sheets, others, such as cytoskeleton proteins and collagens, are naturally insoluble^17^. However, for the majority of other proteins, the reasons behind their tendency to aggregate in aging are unknown. To map the aggregated proteome, bone marrow and splenic cell lysates were fractionated by HF5-UV-MALS and their proteome collected from different fractions according to their (increasing) molecular weight as reported in Fig. 3c. Collected fractions were run on an SDS-PAGE to confirm proper fractionation (Fig. 4a). Spectrometric analysis of protein carbonylation was performed on each fraction and a positive correlation between size of the aggregates and protein carbonylation was observed indicating that over 90% of the carbonylated proteome was present in the aggregates found in the 22 month cell lysates (Fig. 4b).

This prompted us to analyze further the protein aggregates from 22 month cell lysates for carbonyl content. Thus, the high molecular weight fractions 4 and 5 (Fig. 4a) were collected, digested, using a combination of Lys-C and trypsin in 0.1 M bicarbonate buffer and 4 M urea, followed by nanoLC MS/MS analysis of the tryptic peptides (Supplement Table S1a,b,d,e). Among the high molecular weight aggregate proteome (MW > 10^7^) in the spleen and bone marrow, proteins could be mapped to both intracellular and extracellular sources (Fig. 4c and Supplement Table S1a,b,d,e). Gene onthology (GO) annotation grouped the proteins present in the high molecular weight aggregates into a few major pathways, including cellular growth and development, metabolism, cytoskeleton, trafficking and signal transduction (Fig. 4d and Supplement Table S1c,f).

Mass spectrometric analysis of the post-translational modifications mapped to the protein aggregates indicated that lysine and arginine were the most frequently modified amino-acids (Supplement Table S1g,h). In the case of the spleen, lysine was frequently converted to aminoadipic acid while arginine was mostly converted into the glutamic semialdehyde. Monooxidations of lysine, proline, arginine, phenylalanine, histidine and tryptophan were also mapped in the purified 22 months aggregates (Supplement Table 1). Tryptophan was mainly found to be converted into kynurenine and oxolactone while proline was mostly converted to pyrolidone and pyrolidinone (Supplement Table S1g,h).

Similar type of carbonylation was observed in the bone marrow aggregates, albeit compared to the spleen, the bone marrow aggregated proteome showed a lower number of carbonyl moieties (Supplement Table S1g,h).

Altogether, the data confirm an increase tendency of proteins to aggregate during physiological aging^17^ and indicate a positive correlation between protein aggregation and amount of protein post-translational modifications normally associated with chronic oxidative stress.

### *In vivo* Generation of Protein Aggregates through induction of Oxidative Stress

**Figure 5:**
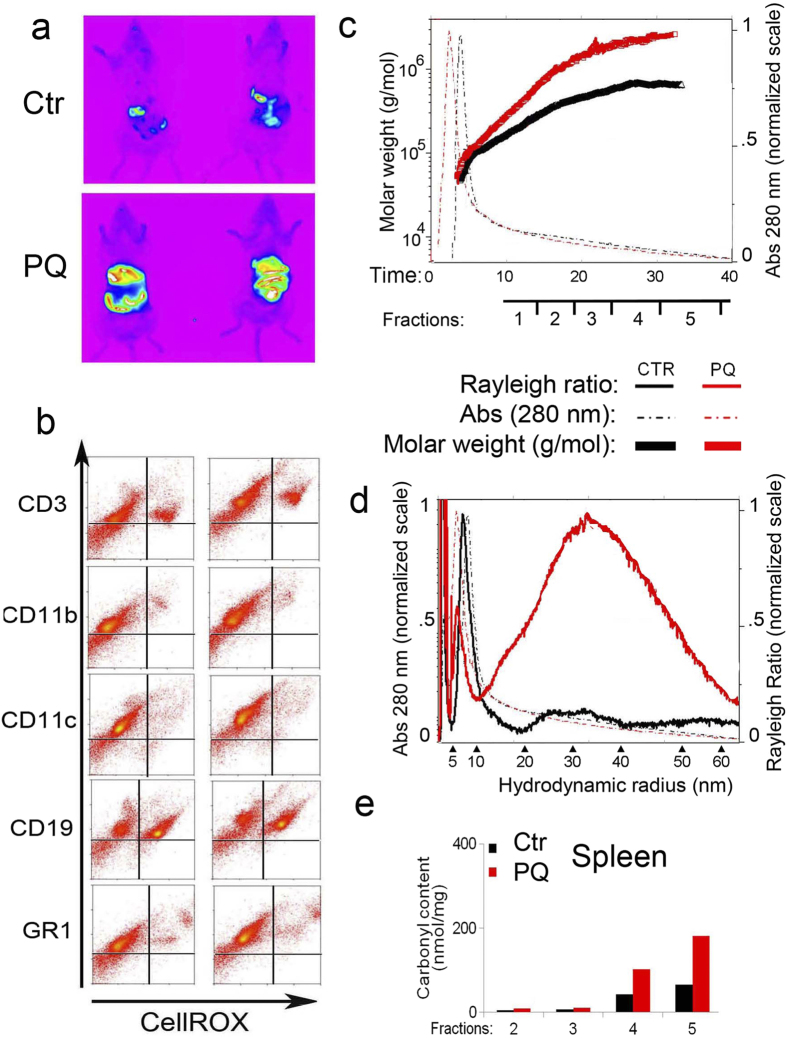
*In vivo* detection and HF5-UV-MALS characterization of oxidatively damaged proteins. **(a)** *In vivo* Detection of Oxidative Stress in control (Ctr) PQ treated mice. Mice were injected i.p with 20 nM PQ for two consecutive days. On day three mice were injected with 2.5 mM of the CellROX probe which fluoresces upon binding to reactive oxygen species. The *in vivo* detection of fluorescence was performed using the *In-Vivo* Imaging System FX PRO (Carestream). One out of three experiments is shown. Three mice (Ctr or PQ-treated) were used in each experiment. **(b)** FACS analysis of splenic cell subpopulations isolated *ex vivo*, following CellROX injection as described in (**a**). One out of three experiments is shown **(c)** HF5-UV-MALS of spleen lysates from control and 10 mM PQ treated mice. Overlaid fractograms: dash-dotted lines report OD reading at 280 nm (right y axis); full thick lines report calculated molecular weight, displayed in logarithmic scale (left y axis); x axis reports the retention time. One out of three runs is shown; each run was performed using cell lysates from independent mice (Ctr and PQ-treated). (**d)** HF5-UV-MALS of spleen lysates from control and 10 mM PQ treated mice. Overlaid fractograms: dash-dotted lines report OD reading at 280 nm (right y axis); thin full lines report Rayleigh ratios at 90° (left y axis); x axis reports hydrodynamic radius values. One out of three runs is shown One out of three runs is shown; each run was performed using cell lysates from independent mice (Ctr and PQ-treated). (**e)** Carbonyl content of protein fractions collected from spleen lysates from control and 10 mM PQ treated mice HF5-fractionated as in (**c**).

As a next step, we asked whether aging-related aggregation could be recapitulated *in vivo* by inducing oxidative stress. To this goal, mice were injected intraperitoneally with Paraquat (PQ) (2.5 mg/25 g body weight) for two consecutive days. The CellRox probe, that fluoresces upon binding to reactive oxygen species, was injected i.v. on day three (Fig. 5a). Increased fluorescence was observed in PQ-treated mice as compared to untreated controls, indicating *in vivo* an increase in oxidative stress (Fig. 5a,b).

Next, total spleen lysates from control and PQ treated mice were submitted to HF5-UV-MALS analysis. The presence of low-abundant, high MW protein aggregates with MWs above 1.2 × 10^6 ^g/mol were observed in PQ-treated spleen lysates, but not in control mice (Fig. 5c,d and Table 2). The PQ–induced protein aggregates amounted to 21.4% of the total protein inside the treated lysate sample (Table 2).

Importantly, quantification of protein carbonylation, as a measure of oxidative stress, determined an increase in the amount of carbonyl groups in each fraction of the PQ-treated mice, but most predominantly in the high MW fractions of the spleen cell lysate (Fig. 5e). Mass spectrometry analysis of the PQ-induced aggregates revealed many of the same modifications previously reported in aging cells (16 and data not shown).

As previously observed in aging-related aggregates, these experimental data confirmed that oxidative stress does induce protein carbonylation and that the modified proteins are most abundantly found in the high MW aggregates^16^.

## Discussion

The folding of proteins into their native state is directly associated with the attainment of the biological function of that protein. The folding process, driven by the cellular chaperones, buries the hydrophobic amino acids into the internal core and exposes the hydrophilic ones to the surface. Any cellular process that interferes with protein folding or which increases unfolding will, by default, increase the exposure of the hydrophobic part of the protein and increase its tendency to aggregate.

Protein aggregation can be observed in several pathological conditions. Mutation in the primary protein sequences, or in chaperones controlling protein folding or processing, is one of the most extensively analyzed causes of protein aggregation. Different familiar forms of neurodegenerative diseases have been associated with mutations that enhance the pro-aggregating properties of specific proteins, such as α-synuclein in Parkinson’s disease^13^,^20^,^21^, tau or beta amyloid in Alzheimer’s^22^,^23^ and huntingtin in Huntington’s disease^24^,^25^,^26^. Additionally, any disease or condition that impairs the proteasome or the endosomal and lysosomal compartments, autophagic clearance or the chaperone folding machinery, will also (by default) increase protein aggregation^1^,^7^,^15^,^27^,^28^,^29^,^30^,^31^,^32^,^33^.

Thus, it has become apparent over the years, that there are several causes that can lead to protein aggregation and that the aggregates themselves may be very different in size, proteomic composition, type of elicited unfolding response, subcellular location, type of clearance and induced biological consequences^1^,^5^,^12^,^24^,^25^,^28^,^29^,^34^,^35^,^36^.

It was previously reported that around 6–9% of the aging proteome in C. elegans has a tendency to aggregate^17^. Our data, using murine bone marrow and splenic cells confirmed this finding, and determined that also in this cell type physiological aging is associated with an increased propensity of the cellular proteome to aggregate^16^. More importantly, we determined that post-translational modifications, normally associated with oxidative stress, are conducive of aggregate formation. Indeed, we mapped increasing amounts of protein modifications by carbonyl groups previously reported to be associated with aging, such as conversion of lysine and arginine to aminoadipic acid and glutamic semialdehyde respectively, tryptophan conversion to kynurenine and oxolactone and proline to pyrolidone and pyrolidinone. Mono and di-oxidations of lysine, proline, arginine, phenylalanine, and histidine were also mapped in the aggregated proteome. The causative role of aging-related oxidative stress in inducing protein aggregation was further determined by PQ treatment, which – by inducing acute oxidative stress – enhanced aggregation of the cellular proteome by producing the same type of carbonyl modifications.

Post-translational modifications including glycation, lipoxidation and carbonylation are often observed in chronic inflammatory and degenerative diseases or metabolic syndrome and diabetes^16^,^34^,^37^,^38^,^39^,^40^,^41^. The nature of these modifications can be different in chronic diabetes, where protein glycation is often observed, to the modifications present in chronic inflammatory conditions, where ROS-mediated carbonylation is the major protein modification^27^,^29^,^37^,^38^,^42^.

Here we report that these modifications are also associated with physiological aging. All carbonylated post-translational modifications are known to form Schiff bases with the free amino groups (especially on Lys, Arg or N-terminal) followed by rearrangements called Amadori products. Both Schiff bases and Amadori products are reversible, with the latter characterized by a higher stability, and thus more prone to generate the final Advanced Glycation End (AGE) products. Furthermore, the carbonylation of side chain amino acids can induce protein aggregation by promoting unfolding and formation of non-covalent (hydrophobic, electrostatics and hydrogen interactions) as well as covalent (Schiff-base adducts) bonds among proteins^34^,^35^.

We also determined that aging-related and oxidative stress-induced aggregates presented with a more compact conformation than the few aggregates present in the cellular proteome of young and adult mice. Indeed, our data indicate that protein carbonylation favors the formation of denser and more compact aggregates. The biophysical properties of the aggregates are important, since it has been shown recently that the autophagic machinery required for the sequestration of the aggregates and their delivery to lysosomes can only assembly on the surface of compact aggregates, whereas those showing active disassembly by chaperones cannot be targeted by this pathway^43^. Additionally, the soluble or insoluble nature of the protein aggregates and their relaxed or compact conformation can have different consequences on cellular proteostasis and toxicity^12^. Soluble and “rod shape” aggregates can be more easily unfolded and disposed of by the proteasome or through chaperone-mediated autophagy, whereas compact aggregates tend to accumulate in the cytosol and compromise cellular functions. Therefore, each type of modification could elicit a different stress response, with recruitment to the aggregate of the ubiquitination machinery or to different types of chaperones that target the aggregate to the proteasome, macroautophagy, microautophagy or chaperone-mediated autophagy according to the aggregate size, conformation, ability to unfold and associated machinery^21^.

At the proteomic level, analysis performed on the highest molecular weight aggregates (MW > 10^7 ^g/mol) confirmed the presence of several proteins previously reported to be insoluble or with increased tendency to aggregate during aging^17^,^18^. Among the former, cytoskeletal proteins, collagens and matrix proteins, are normally insoluble proteins, which partition with aggregates even though their insolubility does not affect their biological functions. Other proteins increase their insolubility with aging, among which, ribosomal proteins, and proteins associated with mitochondrial oxidative phosphorylation and cell growth are highly represented^17^. These proteins have often primary and secondary structures similar to proteins associated with neurodegenerative diseases (poly Q sequence and beta-sheet helices)^13^, or, as reported here, an increase in side-chain carbonyl groups. The presence of carbonyl-related modifications can explain the highly proteomic representation among the aggregates of mitochondrial proteins that are highly prone to oxidative stress^4^.

At the biochemical level, it will be important to gain further insight into the different mechanisms that induce aggregation of the cellular proteome, since it will further our understanding of how cellular proteostasis is compromised at the functional level. For example, in conditions associated with chronic inflammation or prolonged metabolic stress, the aggregates are not induced by protein mutation, but are favored by post-translational modifications, which unfold the proteins and expose their hydrophobic sites^1^,^4^,^34^. Taken together, our analysis confirms increased aggregate formation in the aging proteome and links these phenomena, at least in part, to the increased number of post-translational carbonyl-modifications.

## Methods

### Reagents

Trifluoroacetic acid, acetonitrile, acetic acid, formic acid, iodacetamide and methanol were purchased from Fisher Scientific (Pittsburgh, PA). Urea, thiourea, octylglucoside, dithiothreitol (DTT), iodoacetamide, ammonium bicarbonate, Coomassie Brilliant Blue R-250, KCl, KH_2_PO_4_, H_3_PO_4_ and Na_2_CO_3_ were purchased from SIGMA (St. Louis, MO, USA). Complete Proteinase inhibitor cocktail was also purchased from Sigma. Porcine trypsin (20 ug, specific activity > 5,000 units/mg seq. grade modified) and Lys-C (sequencing grade, 10 ug) were purchased from Promega (Madison, WI). All solutions were prepared using MilliQ water purified by an Elix 3 UV Water Purification System (Millipore, Billerica, USA) and filtered through 0.2 μm pore membrane sterile filter units (Steritop^TM^, Millipore).

### Mice

Female C57BL/6 J mice (3, 12 and 22 months old) were purchased from Harlam as part of the age-controlled NIH mouse colony program. All animal procedures were carried out according to a protocol approved by the Institutional Animal Care of Albert Einstein College of Medicine.

### Cell Preparations

**Bone marrow** cells were flushed from the femur and tibia of 3, 12 and 22 month old mice. Cells were centrifuged at 1500 rpm for 10 minutes. In some experiments CD34^+^ bone marrow precursors were purified by magnetic bead immunoselection (Miltenyi Biotec), following the manufacturer’s instructions. Cells were either plated for 2–3 hours on a cover glass before immunostaining or lysed immediately for biochemical analysis of protein aggregates.

**Spleens** were harvested from 3, 12 and 22 months old C57Bl6 mice, and digested for 30 minutes at 37 °C with 4000 U/ml of collagenase type II (Invitrogen) in sterile Hanks’ balanced saline solution (HBSS) with Ca^2+^ and Mg^2+^ (Life Technologies). Cells were collected through a 70 μm sieve, centrifuged at 1500 rpm for 10 minutes and the cell pellet resuspended in red blood cell lysis buffer (8.3 g/L ammonium chloride in 0.01 M Tris-HCl buffer from Sigma, Aldrich). Following 5 minutes incubation at room temperature, complete media was added to stop the lysis reaction and the cells were pelleted at 1500 rpm for 10 min and further processed for HF5 separation or FACS analysis.

### Induction of oxidative stress

Oxidative stress was induced in C57BL/6 mice by intraperitoneal injection with PQ-saline solution (2.5 mg/25 g body weight) on two consecutive days. Twenty-four hours prior to the experiment, animals were fasted to minimize the potential background. Before imaging, all animals were intravenously injected with Cell-ROX Deep Red (2.5 mM, excitation 640 nm/emission 664 nm, Molecular Probes, CA) for *in vivo* fluorescent imaging using the IVIS (In-Vivo F PRO imaging system from Bruker BioSpin Molecular Imaging, CT) system. Whole animals were imaged 30 minutes post-injection and images were captured at an excitation 610 nm / emission 700 nm using a built-in cooled closed-caption device camera. After a threshold value for background intensity was obtained, different levels of fluorescence intensity were displayed using a pseudo rainbow color scheme, and analyzed using built-in Carestream Molecular Imaging Software.

### Dynamic light scattering (DLS) analysis

The NanoBrook 90Plus Zeta Particle Size Analyzer instrument from Brookhaven Instruments Corporation of Holtsville, New York, USA (particle sizing > 0.3 nm to 6 μm, scattering angle set up at 90°) was used to measure the average diameter and the distribution width (dispersity) of the protein aggregate in bone marrow and spleen cell lysates. Data was analyzed using the built-in ZetaPlus Particle Sizing software 4.11. The following parameters were used to collect the data in the batch mode for all lysates in the same day, after solubilization in the NP-40 cell lysis buffer and adjustment of the protein concentration to 0.5 mg/ml: wavelength laser = 640 nm; angle for scattering = 90^°^; 25 °C; 10 runs for 1 min/each run covering a total time of 10 min for data collection for each sample; solvent = water with the refractive index of 1.330 (real recorded 1.590) and viscosity of 0.890 cP; average count rate = 168.2 kcps; dust filtering = 30.00.

### Size-Exclusion Chromatography

Three hundred micrograms of total spleen lysates from 3, 12 and 22 months old mice were run on a Superdex 300 column at a flow rate of 0.5/ml minute in 1% NP40, 50 mM Tris/HCl, 150 mM NaCl, 5 mM EDTA and 10 mM DTT, supplemented with 1 × protease inhibitor cocktail (Roche). Each column was calibrated with protein markers and blue dextran as molecular weight standards.

### Imunofluorescence

CD34^+^ bone marrow cells were immune-affinity purified, from 3, 12 and 22 months old bone marrows, using the lineage negative selection kit from Miltenyi. Cells were seeded on a collagen-treated cover glass for 2–3 hours (Biocoat). For staining, cells were fixed in 1% paraformaldehyde for 10 minutes at RT, washed twice in 10 mM glycine in PBS and permeabilized for 30 minutes in 0.05% saponin, 0.2% BSA and 0.1% sodium azide in PBS. Cells were stained with the following primary Ab: anti-α tubulin mouse IgG2a (clone YL1) and rabbit polyclonal to ubiquitinated-proteins and inclusion bodies (Ab8134 from Abcam). Secondary antibodies: anti-mouse IgG2a Alexa-Fluor 488 and anti-rabbit-Alexa 568. Fluorescence was viewed with a Leica AOBS scanning Confocal Microscope (Leica).

### HF5-UV-MALS

All separations were performed employing an Agilent 1100 HPLC system (Agilent Technologies, Santa Clara, CA, USA) composed by a degasser, a quaternary pump, an auto sampler and a diode array UV-Vis detector online coupled with an Eclipse® DUALTEC^TM^ flow FFF separation system (Wyatt Technology Europe, Dernbach, Germany). The HF5 channel was equipped with a polyether-sulfone (PES) fiber, type FUS 0181 (Microdyn-Nadir, Wiesbaden, Germany) with the following characteristics: 34 cm length, 0.8 mm inner diameter, 1.3 mm outer diameter, and 10 kDa MWCO, corresponding to an average pore diameter of 5 nm. The HF5 channel employed in this study was the same as that described in a recent publication^19^. The HF5 cartridge assembly and modes of operation of the Eclipse® DUALTEC^TM^ system have already been described in the recent literature^44^.

The ChemStation software, version B.04.03 (Agilent Technologies), was used to operate the HPLC components and the software package Wyatt Eclipse @ ChemStation version 4.02 (Wyatt Technology Europe) was used to run the Eclipse® DUALTEC^TM^ separation system. An 18-angle multi-angle light scattering (MALS) detector, model DAWN EOS^TM^ (Wyatt Technology Corporation, Santa Barbara, CA, USA), with a laser wavelength of 658 nm, was used in all experiments. An Agilent 1100 UV-Vis diode array detector operating at a wavelength of 280 nm was used to detect protein concentration. A second UV signal at 260 nm was registered to assess possible DNA contamination.

Lysates were separated in either native buffer (50 mM Tris-HCl, 150 mM NaCl and protease inhibitors, pH 7.4) or denaturing buffer (50 mM Tris-HCl, 150 mM NaCl, 2 or 4 M urea and proteases inhibitors, pH 8.0). Cell lysates were injected during the focusing step, performed at 0.5 mL/min of focus flow rate for 5 minutes, after which each sample was eluted at 0.5 mL/min of longitudinal flow rate under a field gradient decreasing from 0.4 mL/min to 0.1 mL/min over 70 minutes.

Samples following HF5 separation, the proteome fractions were dialyzed in 10 mM Tris-HCl solution at pH 8.0 using Side–a–lyzer cassettes (MWCO: 3500 Da, 3 – 15 mL, Pierce) or Snake Skin dialysis tubing (MWCO: 3500 Da, capacity of 3.7 mL/cm, Pierce). Fractions were then lyophilized and stored for further analysis.

### Astra Computations

The Astra® software (version 6.0.6, Wyatt Technology Corporation) was used to calculate the MW ranges for all lysate samples; the light scattering intensity registered by detectors 2 through 18 and the concentration signal from the UV detector (280 nm) were correlated through a 1^st^ degree Zimm model, thus providing the MW values. An average value of 1.0 (mL/mg × cm) for the extinction coefficient at 280 nm (ε_280nm_^0.1%^) was used in all MW calculations. The angular dependency of the scattered light was correlated through a 1^st^ degree Zimm model, thus providing the *rms radius* values. The relative amounts of protein aggregates, calculated as % of the total protein present in the cell lysates, were also quantified by Astra. Astra software was also employed to determine the molecular conformation of the protein aggregates. The correlation plots (conformation plots) were generated from experimental data, by plotting the *rms radius* values against the corresponding MW values (both in logarithmic scale). The plots were fitted linearly and the slope values were assigned to known conformation types. A slope value of 1.0 corresponds to a *rod* conformation, a slope value of 0.5–0.6 corresponds to a *random coil* conformation and a slope value of 0.33 corresponds to a *sphere* conformation. The trend (slope and/or change of the slope) of the molecular conformations was determined for *rms radius* values above 10 nm (due to DAWN EOS® instrumental range limitation), with corresponding MW values usually above 1 × 10^6 ^g/mol.

### Hydrodynamic radius (*r*_*h*_) calculations

The Eclipse ISIS simulation software (Superon GmbH) was employed for the calculation of the *r*_*h*_ range for each cell lysate (reported in each graph as a secondary x axis), based on the experimental retention time (*t*_*R*_) values and inputting the HF5 method flow rates. The viscosity values for the carrier solutions were extrapolated from data found in literature^45^ (η_water_,_25 °C_ = 10^−3 ^N·s/m^2^ for Tris-HCl buffer; 1.0909 × η_water_,_25 °C_ for Tris-HCl + 2 M urea and 1.2215 × η_water_,_25 °C_ for Tris-HCl + 4 M urea).

### Cell Staining and FACS analysis

C57Bl6 spleen cells and bone marrow were harvested from control and PQ-treated mice, previously injected with Cell-ROX Deep Red. Spleens were digested for 30 minutes at 37 °C with collagenase (4000 U/ml collagenase type II from Invitrogen) in sterile Hanks’ balanced saline solution (HBSS) with Ca^2+^ and Mg^2+^ (Life Technologies). Cells were then collected using a 70 μm sieve, centrifuged at 1500 rpm for 10 minutes and the cell pellet resuspended in red blood cells lysis buffer (Sigma) for 5 min at RT. Cells were then washed at 1500 rpm for 10 minutes and the pellet resuspended in FACS buffer (PBS supplemented with 4% FBS and 0.2% sodium azide). The following FITC labeled antibodies were used for staining: CD3 (clone 17A2, Cat#561798, Pharmingen), CD19 (clone 1D3, Cat#557399, Pharmingen), CD11b (cloneM1/70 Cat#557672, Pharmingen), CD11c (clone HL3 Cat#553801, Pharmingen), and GR-1 (clone RB6-8C5, Cat#553127, Pharmingen. Cells (0.5 × 10^6^) were incubated for 30 minutes on ice with saturating amount of antibodies in staining buffer (2% FBS, 5 mM EDTA in DPBS). After 2 washes, the cells were analyzed using the LSR2 (Becton Dickinson, N.J, USA).

### SDS-PAGE and Native gel

Equal amounts (10–25 μg) of proteins (including the lyophilized aggregates fractions) were boiled and reduced with beta-mercaptoethanol in the 4× SDS sample buffer and run on Mini-PROTEAN® TGX^TM^ Gels (4–15%, BioRad, CA). Gels were silver stained using the Pierce Color Silver Stain Kit (Pierce).

### Western blot analysis of carbonyl groups

In some experiments (Fig. 1e) primary cell lysates were derivatized using the Oxyblot Protein Oxidation Detection Kit (Millipore, USA), separated on 4–15% SDS-PAGE, and the transferred membranes were incubated with a rabbit polyclonal anti-DNPH antibody followed by a goat anti-rabbit IgG–HRP antibody (OxiSelect™ Protein Carbonyl Immunoblot Kit (STA-308) from Cell Bioloabs, Inc). Proteins were visualized by chemiluminescence.

In other experiments (Fig. 3a) cells were lysed in RIPA buffer supplemented with 1 × protease inhibitor cocktail (Roche) for 30 minutes, on ice. Lysates were spun twice at 20,000 g to collect the soluble proteins present in the supernatant. The pellet was resuspended in 4 M urea, 2% SDS, 50 mM DTT, 50 mM Tris pH 7.4 at RT before loading on SDS-PAGE.

### Spectrophotometric determination of protein carbonyl content

The carbonyl content of the purified aggregates was quantified spectrophotometrically with the Protein Carbonyl Assay kit (OxiSelect™ Protein Carbonyl Spectrophotometric Assay kit STA-315 from Cell Biolabs Inc.).

### Proteomic Analysis of HF5 purified aggregates

Aggregates purified by HF5 (High MW fractions from 22 month spleen and bone marrow) were incubated in 0.1 M ammonium bicarbonate buffer with 4 M urea and sonicated for 15 minutes at RT. The samples were further reduced with 10 mM TCEP-HCl in 0.1 M ammonium bicarbonate buffer, pH 8.9, for 30 minutes, at RT. The reduction was followed immediately by alkylation with 55 mM iodacetamide solution in 0.1 M ammonium bicarbonate, at RT, in the dark for 45 minutes. Proteins were digested with trypsin and Lys-C (sequencing grade, Promega) at 37 °C for 12 hours. Following digestion, the samples were subjected to desalting using ZipTip (C18) reversed-phase system (Millipore, Billerica, MA) and submitted for MS/MS peptide sequencing.

### NanoLC-ESI-MS/MS Analysis of Tryptic Peptides

LTQ-MS/MS sequencing was performed using a Nanospray LC-MS/MS on a LTQ linear ion trap mass spectrometer (LTQ Thermo Scientific, San Jose, CA, USA) interfaced with a TriVersa NanoMate nanoelectrospray ion source (Advion BioSciences, Ithaca, NY, USA). The 15 most abundant ions were selected for MS/MS.

#### Database searching

Tandem mass spectra (raw files) were extracted and converted to mgf files using Proteome Discoverer 1.3 (Thermo Fisher Scientific). All MS/MS mgf were analyzed using Mascot (Matrix Science, London, UK; version 2.5.0.1). Mascot was set up to search the SwissProt_AC_2014_11 database (selected for Mus musculus, unknown version, 16696 entries). Mascot was searched with a fragment ion mass tolerance of 0.50 Da and a parent ion tolerance of 1.5 Da. Carbamidomethyl cysteine (+57 on C) was specified in Mascot as a fixed modification. The following chemical modifications were specified in Mascot as variable modifications: Arg- > GluSA of arginine (−43 on R), pro- > Pyrrolidinone of proline (−30 on P), pro- > Pyrrolidone of proline (−28 on P), deamidation of asparagine and glutamine (+1 on NQ), trp- > Kynurenin of tryptophan (+4 on Trp), pro- > pyro-Glu of proline (+14 on P), trp- > Oxolactone of tryptophan (+14 on W), trp- > hydroxykynurenin on tryptophan (+20 on W), M- > Dioxidation on methionine (+16 on M), Glu- > pyro-Glu of glutamate (−18 on E), Gln- > pyro-Glu of glutamine (−17 on Q) and lys- > AminoadipicAcid of lysine (+15 on K). In addition, monoxidation on any of the C,D,F,H,K,M,N,P,R,W,Y (+16 Oxidation) was also specified as a variable modification. The 4-hydroxynonenal (HNE) was specified as a variable modification for any of the C, N, K (+156 on CHK) for searching the advanced lipooxidation end products. Contaminants, such as keratins were removed and a false discovery rate (FDR) for peptide identification was assessed by decoy database searching and was finally adjusted to less than 1.5% for proteins and peptides.

#### Criteria for protein identification

Scaffold (version Scaffold_4.3.4, Proteome Software Inc., Portland, OR) was used to validate MS/MS based peptide and protein identifications. The Mascot DAT files for spleen and bone marrow samples were loaded in Scaffold using the built in “ MuDPIT” option. Peptide identifications were accepted when they attained greater than 95.0% probability by the Scaffold Local FDR algorithm. Protein identifications were accepted when they attained greater than 90.0% probability and contained at least 1 identified peptide. Protein probabilities were assigned by the Protein Prophet algorithm. The FDR was adjusted to less than 1% for peptides and less than 5% for proteins (p < 0.05). Proteins that were identified by unique “red-bolded” peptide had their tandem MS spectra checked manually. Proteins that contained similar peptides and could not be differentiated based on MS/MS analysis alone were grouped to satisfy the principles of parsimony. Proteins sharing significant peptide evidence were grouped into clusters. Proteins were annotated with GO terms from NCBI (downloaded Mar 18, 2015).

#### Calculation of PTM in the proteome of aggregates

The specific oxidative PTMs found in each proteomic expression profile from the bone marrow and spleen are reported as % from the total number of PTM sites (excluding the variable modification “deamidation” and the fixed modification “carbamidomethyl) using as total the number of chemical modification reported in Supplemental Table 1. Only the PTMs characterized by > 95% localization probability were taken into account.

### Statistical Analysis

Statistical analysis was performed using Windows GraphPad Prism 6 (GraphPad Software, La Jolla, California, USA). One way ANOVA, using Dunnett’s multiple comparisons test, was used to assess the statistical significance between the mean values of each sample group.

## Additional Information

**How to cite this article**: Tanase, M. *et al.* Role of Carbonyl Modifications on Aging-Associated Protein Aggregation. *Sci. Rep.* **6**, 19311; doi: 10.1038/srep19311 (2016).

## Supplementary Material

Supplementary Information

Supplementary Table S1

## Figures and Tables

**Table 1:** Analysis of protein aggregates in bone marrow cell lysates from 3, 12 and 22 months old mice under *native* and *denaturing* conditions.

Bone marrow cell lysates			
Analysis of Proteins Fractionated in Tris-HCl			
Sample	3 months	12 months	22 months
MW range: common proteome (g/mol)	5.0 × 10^4^ – 4.4 × 10^6^		
Hydrodynamic radius range (nm) of The common proteome	3–111	3–111	3–53
MW range of aging – induced aggregates (g/mol)	–	–	4.4 × 10^6^–1.3 × 10^7^
Hydrodynamic radius range (nm) of aging – induced aggregates	–	–	53–111
Aging – induced aggregates as % of the total proteome	–	–	10.9%
**Analysis of Proteins Fractionated in Tris–HCl + 2 M urea**			
Common MW range (g/mol)	1.0 × 10^5^–4.4 × 10^6^		
Hydrodynamic radius range (nm)	4–90	4–89	4–66
MW range of aging–induced aggregates (g/mol)	–	–	4.4 × 10^6^–1.3 × 10^7^
Hydrodynamic radius range (nm) of aging – induced aggregates	–	–	66–97
Amount of aging – induced aggregates	–	–	**11.5%**
MW range of new aggregates (denaturing conditions)	4.4 × 10^6^–1.2 × 10^7^	4.4 × 10^6^–1.2 × 10^7^	1.3 × 10^7^–2.1 × 10^7^
Hydrodynamic radius range (nm) of new aggregates	90–113	89–113	97–113
Amount of new aggregates (denaturing conditions)	**2%**	**3%**	**12%**
**Analysis of Proteins Fractionated in Tris–HCl + 4 M urea**			
Common MW range (g/mol)	1.0 × 10^5^–4.4 × 10^6^		
Hydrodynamic radius range (nm)	3–7	3–7	3–9
MW range of aging – induced aggregates (g/mol)	–	–	4.4 × 10^6^–1.3 × 10^7^
Hydrodynamic radius range (nm) of aging – induced aggregates	–	–	9–18
Amount of aging – induced aggregates	–	–	11%
MW range of new aggregates (denaturing conditions)	4.4 × 10^6^–3.0 × 10^7^	4.4 × 10^6^–3.0 × 10^7^	4.4 × 10^6^–1.5 × 10^7^
Hydrodynamic radius range (nm) of new aggregates	7–51	7–51	18–51
Amount of new aggregates (denaturing conditions)	49%	56%	7%

**Table 2:** Analysis of proteins aggregates, fractionated from spleen lysates harvested from controls and PQ–treated mice.

Spleen cell lysates					
Sample	MW range	Hydrodinamic radius range (nm)	MW range of PQ – induced aggregates (g/mol)	Hydrodinamic radius range of PQ-induced aggregates (nm)	% of PQ– induced aggregates
Control	5.0 × 10^5^–1.2 × 10^6^	2–55	–	–	–
Treated (10PQ)	5.0 × 10^5^–1.2 × 10^6^	2–23	1.2 × 10^6^–2.6 × 10^6^	23–55	**21.4%**
