# Supplementary material for: Role of Carbonyl Modifications on Aging-Associated Protein Aggregation
Source: Sci Rep. 2016 Jan 18;6:19311. doi: 10.1038/srep19311 (PMC4726109; doi:10.1038/srep19311)
Supplement: Supplementary Information [file srep19311-s1.pdf]

Role of Carbonyl Modifications on Aging-Associated Protein Aggregation

Maya Tanase, Aleksandra M. Urbanska, Valerio Zolla, Cristina C. Clement, Liling Huang, [1]Kateryna Morozova, Carlo Follo, Michael Goldberg Barbara Roda, Pierluigi Reschiglian, [1,4,5] Laura Santambrogio.

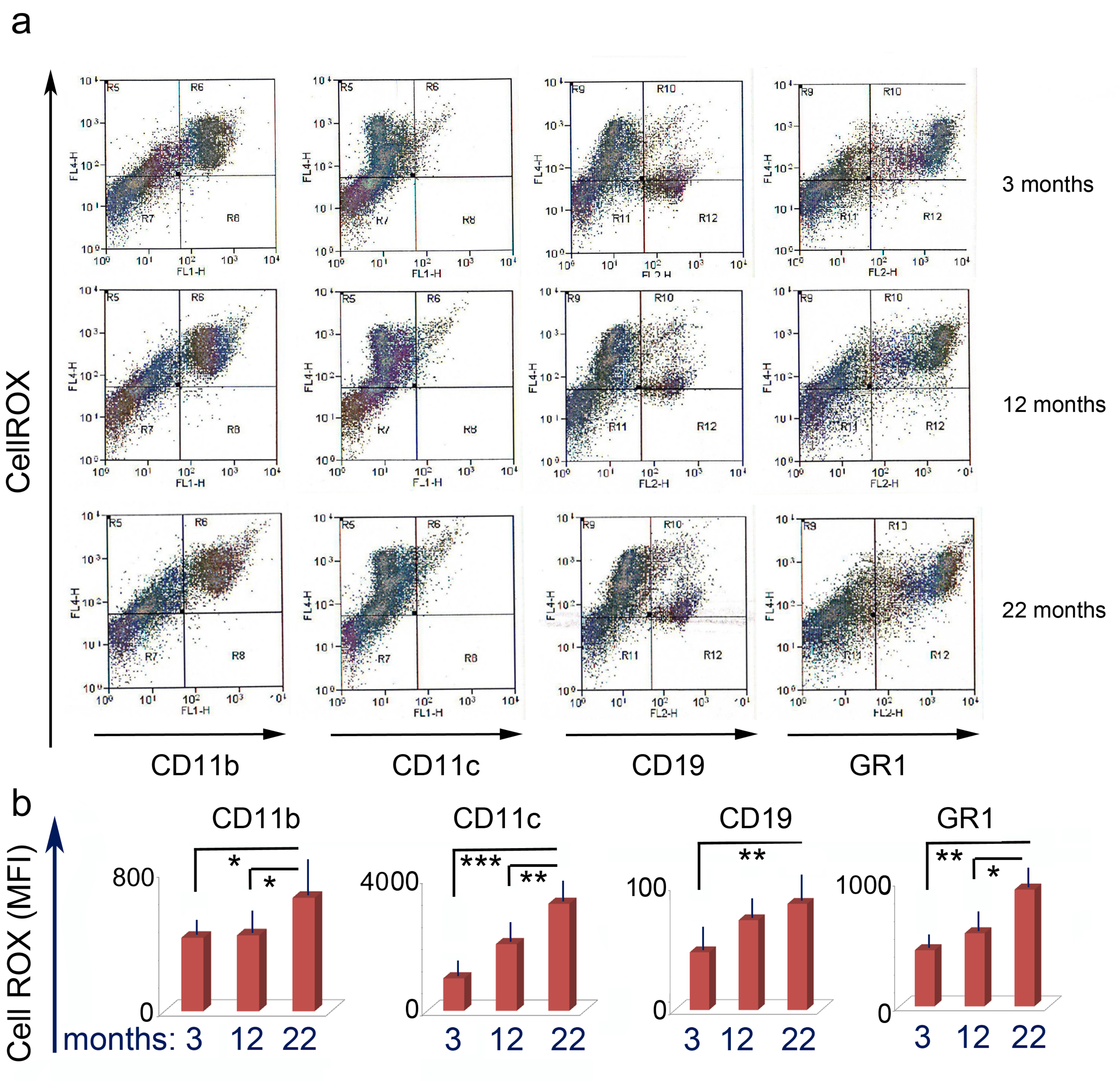
a
CellROX
3 months
12 months
22 months
CD11b
CD11c
CD19
GR1
b
Cell ROX (MFI)
CD11b
CD11c
CD19
GR1
months: 3 12 22

a) FACS analysis of bone marrow cell subpopulations isolated *ex vivo*, following CellROX injection in the same mice imaged in (a). **b)** Bar graphs representing CellROX mean fluorescence index of splenic cell subpopulations in 3, 12 and 22 months old mice. Average and standard deviation calculated from three separate experiments, each experiment included two independent mice for each age group. * $p < 0.05$ ** $p < 0.01$
